# Deep learning for real-time single-pixel video

**DOI:** 10.1038/s41598-018-20521-y

**Published:** 2018-02-05

**Authors:** Catherine F. Higham, Roderick Murray-Smith, Miles J. Padgett, Matthew P. Edgar

**Affiliations:** 1 School of Computing Science, University of Glasgow, Glasgow, G12 8QQ UK; 2 School of Physics and Astronomy, University of Glasgow, Glasgow, G12 8QQ UK

## Abstract

Single-pixel cameras capture images without the requirement for a multi-pixel sensor, enabling the use of state-of-the-art detector technologies and providing a potentially low-cost solution for sensing beyond the visible spectrum. One limitation of single-pixel cameras is the inherent trade-off between image resolution and frame rate, with current compressive (compressed) sensing techniques being unable to support real-time video. In this work we demonstrate the application of deep learning with convolutional auto-encoder networks to recover real-time 128 × 128 pixel video at 30 frames-per-second from a single-pixel camera sampling at a compression ratio of 2%. In addition, by training the network on a large database of images we are able to optimise the first layer of the convolutional network, equivalent to optimising the basis used for scanning the image intensities. This work develops and implements a novel approach to solving the inverse problem for single-pixel cameras efficiently and represents a significant step towards real-time operation of computational imagers. By learning from examples in a particular context, our approach opens up the possibility of high resolution for task-specific adaptation, with importance for applications in gas sensing, 3D imaging and metrology.

## Introduction

A single-pixel camera captures images by temporally measuring the unique spatial properties of a scene with a single detector, rather than using a multi-pixel sensor^1–5^. For this approach a lens is used to form an image of the scene onto a spatial light modulator (SLM), which scans a known basis and produces a vector of encoded intensities measured on a single-pixel detector. A computer algorithm is used to solve the inverse problem to reconstruct an image. This technique enables a solution for applications when conventional imaging with a multi-pixel sensor is not possible or prohibitively expensive, such as at shortwave-infrared and terahertz wavelengths, as well as three-dimensional ranging.

It is important to recognise that there are two time-penalties associated with single-pixel imaging: the acquisition time and the reconstruction time. These become important considerations when moving to higher image resolutions and when applications demand faster frame rates. The acquisition time is determined by the number of unique measurements required and the rate at which this is achieved (the modulation rate of the SLM), whereas the reconstruction time is the computational overhead associated with the complexity of the image reconstruction problem and its implementation.

One of the challenges with single-pixel imaging is overcoming these time penalties. In recent years there has been a wealth of research on the development of various compressive (compressed) sensing strategies that allow for fewer measurements than the number of pixels by exploiting prior knowledge about the scene properties. However, reducing the acquisition time requires solving an under-determined problem via optimisation, which can lead to an increase in the reconstruction time. In many cases the reconstruction time exceeds the acquisition time which prevents real-time applications.

Deep neural networks are computational models which are concerned with learning representations of data with multiple levels of abstraction. They are proving very successful at discovering features in high-dimensional data arising in many areas of science^6,7^. Recently, there has been interest in using deep neural networks to recover structured signals (in particular, images) from their under-sampled random linear measurements^8^. Specifically, dimension reduction in high-dimensional data can be achieved by training a multilayer neural network called an auto-encoder^9^. Innovations such as convolutional layers^10^ have further improved context learning and super-resolution^11^. Thus an auto-encoder can be considered as a promising alternative to compressive sensing. This insight motivated the work presented here, which demonstrates the use of a deep convolutional auto-encoder network (DCAN) to provide a computationally-efficient and data-efficient pipeline for solving the inverse problems with better quality, and importantly, in real-time.

In this work we demonstrate the application of deep learning with a deep convolutional auto-encoder to produce a novel algorithm capable of recovering real-time high-resolution (128 × 128 pixel) video at 30 fps from a single-pixel camera system employing, as a spatial light modulator, a high-speed digital micro-mirror device (DMD). The customized deep learning framework includes a specifically adapted encoding layer equivalent to performing spatial filtering with a chosen binary basis. We further utilise the recovery framework to optimise a highly-compressed measurement basis, which yields better quality images compared with other methods attempting real-time image reconstruction.

## Results

### Experimental set-up

**Figure 1:**
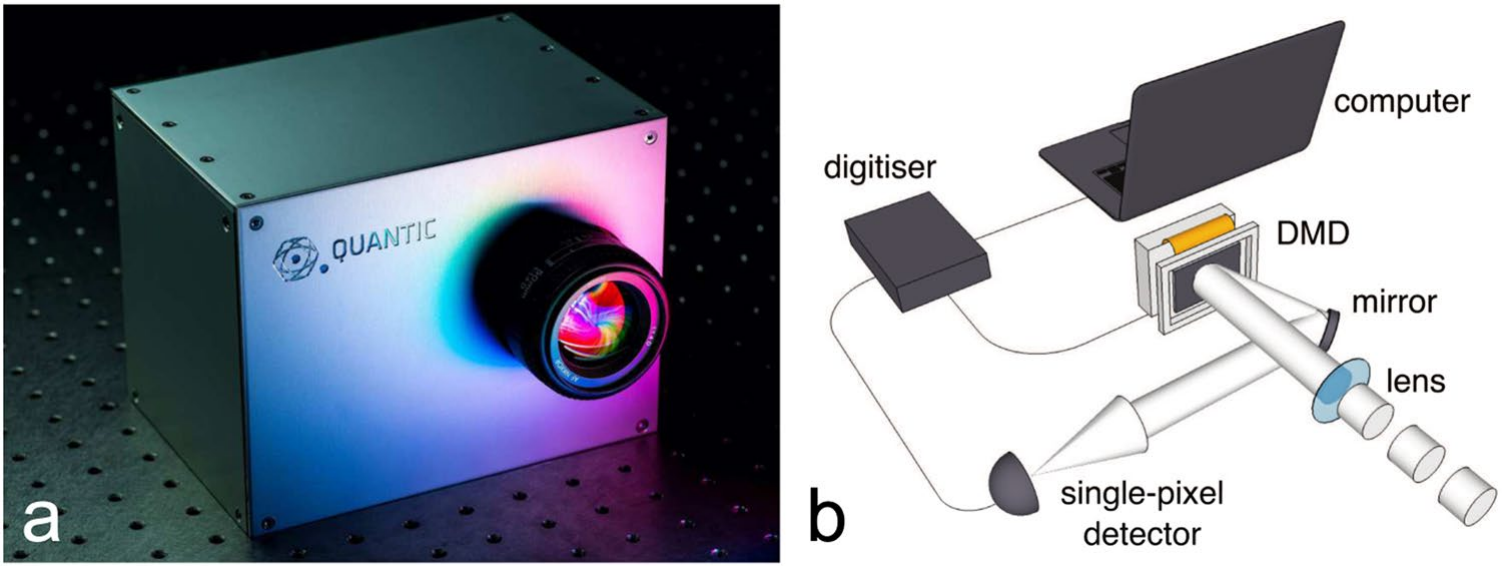
The experimental set-up used for real-time video with a single-pixel detector. A photograph of a single-pixel camera system developed by the Optics Group at the University of Glasgow. The photograph was taken by Kevin Mitchell (**a**). On the right is a simplified schematic of the inner components consisting of a lens, DMD, single-pixel detector, analogue to digital converter and computer (**b**). The lens is used to form an image of the scene onto the DMD, which spatially modulates the light using a series of patterns derived by the method described in the text, and the reflected light intensity is measured on single-pixel detector whereupon the signal is digitised by an analogue-to-digital converter for subsequent computer processing and image reconstruction.

The single-pixel camera, illustrated in Fig. 1, consists of a lens to form an image at the focal plane where a high-speed DMD is located instead of a multi-pixel sensor. The DMD (Vialux V7000 module employing a Texas Instruments 4100) is used to sequentially scan through a series of binary patterns, each of which masks some areas of the image while reflecting the light in other areas onto a single-pixel detector (Thorlabs photomultiplier tube, model PMM01) via a beam steering mirror. The intensities measured for the entire basis are digitised using an analogue-to-digital converter (National Instruments, model USB-6210) whereupon subsequent signal/image processing is performed by computer. In other words, for each binary mask displayed on the DMD, the measured intensity corresponds to the correlation it has with the scene.

### Scanning strategies

The Hadamard basis, which has been used extensively in similar computational imaging schemes, is a set of orthonormal binary matrices with elements that take the value of +1 or −1^12,13^. This basis, which transmits and masks equal proportions of the image, has been shown to improve the signal-to-noise ratio for measurements made by the detector and consequently improves the overall reconstructed image quality. Furthermore a fast algorithmic transform implementation for the Hadamard basis^14^ enables a computationally efficient reconstruction by reducing the number of operations for reconstructing an image of size *N* = *n* × *n* from *N*^2^ to *N* log_2_*N*. However to fully sample an image of this size requires the use of *N* patterns.

In this work we consider several strategies to substantially reduce the number of Hadamard patterns used for scanning the spatial properties of images: randomly ordered (*rand*), Russian-doll ordered (*RH*)^15^ and a novel optimised ordering (*OH*). The random ordering picks rows at random from the Hadamard basis for reshaping and displaying on the DMD. The Russian-doll ordering is an arrangement such that at discrete increments complete sampling is obtained for different spatial resolutions. The optimised ordering is obtained by simulating the signals (correlation strength) from a diverse set of 20,000 training images^16^ and ordering according to their magnitudes in descending order. We also propose a novel non-orthonormal basis, which is generated and optimised by our deep learning approach.

### Reconstruction algorithm and optimised binary basis using deep learning

**Figure 2:**
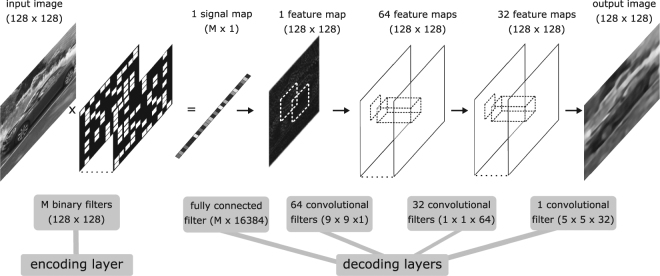
The deep convolutional auto-encoder network (DCAN) architecture. The DCAN is a computational model, comprising encoding (measurement) and decoding (reconstruction) layers, whose objective is to reproduce an input image or scene as an output image. These layers are essentially mappings defined by filters which pass or convolve over the feature maps with strides of one horizontally and vertically. The input scene is measured or encoded by *M* binary filters 128 × 128 (for 30 Hz or 15 Hz frame rates *M* = 333 or *M* = 666 respectively) and reconstructed or decoded using a fully connected layer and a series of three convolutional blocks. After the fully connected layers is a normalisation layer and between the convolutional layers is a non-linear activation layer, not shown here. The DCAN is trained using a large library of images. During training the parameter values (filter weights) are optimised using the stochastic gradient descent method with respect to minimising a standard cost function measuring the Euclidean distance between the predicted output and the desired output.

We designed and trained a deep convolutional auto-encoder network (DCAN) to compress (encode) and decompress (decode) an image for single-pixel camera application. The DCAN comprises a series of filters and activations, referred to as layers, which map input features to output features. The number and shape of the filters and the type of mapping form the architecture of the auto-encoder, shown in Fig. 2. The *M* filters associated with the first *encoding layer* or *bottleneck layer* are designed to mimic the application-specific action of the DMD and are constrained during training to take binary values. A digital image filtered by this binary layer produces a grayscale output in the same way that the patterns loaded on to the DMD filter a scene to produce a light intensity signal. The role of the subsequent *decoding layers* is to reconstruct the image from the compressed signal. The layers of the DCAN include convolutional filters which pass over the feature map(s) providing neighbouring pixel context to the reconstruction. The number and shape of these filters in terms of height, width and depth are chosen to improve the resolution of the output and were informed by the super resolution work by Dong *et al*.^11^. Three convolutional decoding layers are used, with filter dimensions (9 × 9 × 1), (1 × 1 × 64) and (5 × 5 × 32) respectively. A relatively large kernel size of (9 × 9 × 1) in the first convolutional layer was found to provide slightly better results than (5 × 5 × 32) for a resolution size 128 × 128. The filter weights (parameters) were optimised by training the DCAN over a large database of images^16^ (further details in Methods).

**Figure 3:**
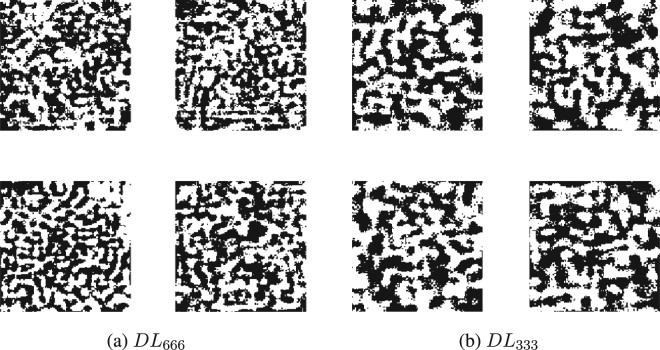
An illustrative selection of deep learned binary patterns. Four randomly chosen patterns, for the same resolution of image, arising when *M*, the number of patterns, is reduced to 666 (**a**), and when *M* = 333 (**b**). These pattern formations suggest that when there are fewer patterns available, the algorithm focuses on low-resolution bases.

There are two types of outputs from training the DCAN. First, a fully parametrised multi-layered mapping algorithm provides a novel inverse method to reconstruct an image from a series of spatially encoded measurements. Second, the *encoding layer*, i.e. the binary sampling basis, can be optimised to yield best reconstruction performance. One approach is to learn just the reconstruction mapping using the novel optimised Hadamard ordering for the encoded measurements (*DL*_*OH*_). Another approach is to learn a binary sampling basis, optimised along with the reconstruction (*DL*). A random sample of deep learned binary patterns for two *M* values resulting from this latter, all encompassing, approach are provided in Fig. 3.

With our single-pixel camera the maximum modulation rate of the DMD is 22.7 kHz but to avoid unwanted noise resulting from micro-mirror relaxation we chose to operate the DMD at 20 kHz. Moreover, since our single-pixel detector is sensing the light from only one output port of the DMD, after each pattern we display the corresponding negative pattern, and take the difference of the two signals to obtain a differential measurement appropriate for a binary basis with values of +1 and −1. Motivated to achieve real-time video-rate image reconstruction of 30 fps or 15 fps this allowed for a maximum of 333 or 666 patterns respectively. We experimentally performed sub-sampling of scenes to this degree using the aforementioned basis orderings whilst also applying three different image reconstruction approaches, and compared the results quantitatively.

### Image reconstruction

Compressive sensing and ${\ell }_{1}$ minimisation^17–19^ provides a theoretical basis for recovering an image from a set of under-sampled measurements by solving a convex optimization program and is commonly used in image reconstruction. Another method used for comparison was the fast Hadamard transform^12^. The final approach utilised the deep learned auto-encoder neural network. (See Methods for further details about image reconstruction).

### Simulation

**Figure 4:**
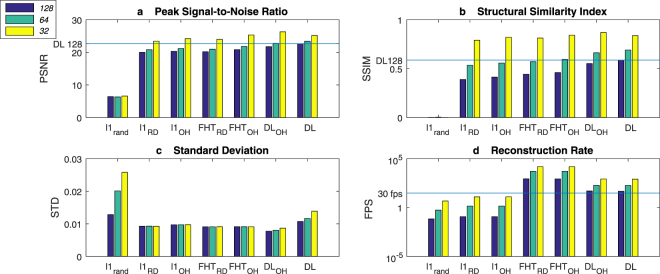
Quantitative assessment of the reconstruction performance. Four different metrics have been used to assess the reconstruction performance (PSNR, peak signal-to-noise ratio (**a**), SSIM, structural similarity index (**b**), STD, standard deviation (**c**) and reconstruction rate (**d**)) using different reconstruction methods: compressive sensing *l*_1_ minimisation using a pseudo-random basis (*l*_1r*and*_), a Russian-Doll ordered Hadamard basis (*l*_1*RD*_), an optimised order of the Hadamard basis (*l*_1*OH*_); by performing a Fast Hadamard Transform when using the Russian-Doll ordered Hadamard basis (*FHT*_*RD*_), the optimised order of the Hadamard basis (*FHT*_*OH*_); as well as the novel methods proposed here employing deep learning (*DL*_*OH*_ and *DL*). In each case the three shaded bars depict different reconstruction resolutions 128 × 128 *first column*, 64 × 64 *second column* and 32 × 32 *third column*. In summary, we find that deep learning offers the best reconstruction quality, with an increase in the standard deviation at video rates.

To understand the expected performance for each of the scanning and reconstruction strategies (*l*1_*rand*_, *l*1_*RD*_, *l*1_*OH*_, *FHT*_*RD*_, *FHT*_*OH*_, *DL*_*OH*_ and *DL*) we simulated noisy signal data for one hundred images selected randomly from the 10,000 images from the image library which were not used for training. Gaussian noise, at an equivalent magnitude to the experimentally measured detector noise (see Fig. 7), was added to the clean signal. Three different metrics were chosen to quantitatively assess the reconstructions: peak-signal-to-noise ratio (PSNR), structural similarity index (SSIM) for a human perception perspective^20^ and standard deviation (SD). We also estimate the reconstruction rate for each method. Due to the excessive running time of the *l*1 method, we evaluate this method on 5 images instead of 100. Choosing to display 333 unique patterns yields a reconstruction frame rate of 30 Hz for image resolution sizes of 32 × 32 pixels (67% compression), 64 × 64 pixels (92% compression) and 128 × 128 pixels (98% compression). The results are shown in Fig. 4 and Table 1.

**Table 1:** Comparison of reconstruction performance for M = 333 patterns. In each cell the two values correspond to PSNR and SSIM respectively.

resolution	*l*1_*rand*_	*l*1_*RD*_	*l*1_*OH*_	*FHT*_*RD*_	*FHT*_*OH*_	*DL*_*OH*_	*DL*
number of images	5	5	5	100	100	100	100
128 × 128	6.4, 0.001	20.0, 0.389	20.3, 0.413	20.2, 0.442	20.8, 0.459	21.7, 0.551	**22.7**, **0.587**
64 × 64	6.3, 0.003	20.8, 0.535	21.2, 0.557	21.0, 0.571	21.8, 0.594	22.8, 0.663	**23.4**, **0.691**
32 × 32	6.6, 0.000	23.4, 0.790	24.2, 0.821	24.0, 0.813	25.3, 0.842	**26.3**, **0.869**	25.2, 0.838

From these simulations we observe that the deep learned approaches, *DH*_OH_ and *DL*, perform best across all resolutions in terms of both PSNR and SSIM, with the 128 × 128 resolution (98% compression) having the largest difference, particularly for SSIM. Learning the binary sampling basis, *DL*, further enhances performance at the higher compression levels, resolutions 128 × 128 and 64 × 64. We also note that comparing the different sampling bases with each reconstruction method, the optimised Hadamard ordering performs better than Russian-Doll ordering. The presented *FHT* and *l*1 reconstructions use a different image base in terms of number (100 for *FHT* and 5 for *l*1) but further investigation on the same image base confirm the indication shown here that *FHT* performs slightly better than *l*1 on the PSNR and SSIM metrics. Importantly, the reconstruction rates are orders of magnitude faster for *FHT* than *l*1 minimisation, and furthermore the deep learned reconstruction rate is comfortably above the desired 30 fps. Thus, in summary, we find that deep learning offers the best reconstruction quality, with an increase in the standard deviation at video rates.

### Experimental results

To obtain experimental results a single large area photomultiplier (Model Thorlabs PMM-01) was placed at an output port of the DMD as depicted in Fig. 1. To help remove noise on the measured signals due to fluctuations in ambient light levels, we choose to display each pattern and its inverse in succession, from which we obtain a series of differential intensities by subtracting one signal from the other. The differential signals were fed forward through the learnt DCAN reconstruction algorithm producing an output image (128 × 128) for each frame of acquisition. We obtained experimental results for *M* = 333 and *M* = 666, yielding a real-time video rate at 30 Hz and 15 Hz respectively.

**Figure 5:**
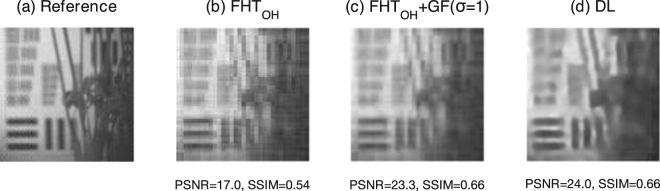
Single-Pixel Camera Performance Results. Comparing the full Hadamard basis (*M* = 16834 patterns) (**a**), with the reduced optimised Hadamard basis (*M* = 666 patterns, 4% of total) (**b**), the reduced optimised Hadamard basis smoothed using a Gaussian filter with 1 pixel standard deviation (*M* = 666 patterns) (**c**) and the deep learned basis (*M* = 666 patterns) (**d**). Note the improved sloping lines in *DL* compared to *FHT*.

As we are interested in comparing different approaches that can achieve real-time video rates, we limit our comparison to the Fast Hadamard Transform and our proposed DCAN reconstruction algorithm. In one experiment, the scene under investigation consisted of a large area USAF test target (300 mm × 300 mm) in the background and a potted plant in the foreground. Performing a fast Hadamard transform with the complete basis (M = 16384) and averaging over 100 frames yields a reference for comparison purposes, see Fig. 5(a). Performing a Fast Hadamard Transform using only a subset of 666 Hadamard patterns (96% compression), the reconstruction exhibits resolution-limited features but with reasonable contrast, as shown in Fig. 5(b). The reconstruction using the *M* = 666 optimised binary basis and the DCAN reconstruction algorithm is shown in Fig. 5(d), which results in more natural features and reduces the apparent noise. To verify that the deep learning method is doing more than simply smoothing over the data, we can apply a Gaussian smoothing Kernel (with 1 pixel standard deviation) to Fig. 5(b) which results in Fig. 5(c). We find that the DL reconstruction compares similarly to the other methods regrading horizontal and vertical features, since the optimised Hadamard order contains predominantly low resolution patterns, however diagonal features and natural shapes appear to be recovered with better sharpness and contrast.

Undertaking a quantitative assessment on the experimental results using the PSNR and SSIM metrics with the reference image, we obtain superior results for the *DL* approach compared to the *FTH*_*OH*_ without smoothing in terms of PSNR (24.0 and 17.0 respectively) and SSIM (0.66 and 0.54 respectively). Smoothing the *FTH*_*OH*_ does improve the quantitative results (PSNR = 23.3 and SSIM = 0.66) but qualitatively, visual inspection suggests an over-smoothed appearance, see Fig. 5. These results validate the conclusion that the deep learning algorithm and optimised sampling basis outperform the other techniques investigated and importantly can be implemented in real-time on a standard computer processor (model Intel i7).

**Figure 6:**
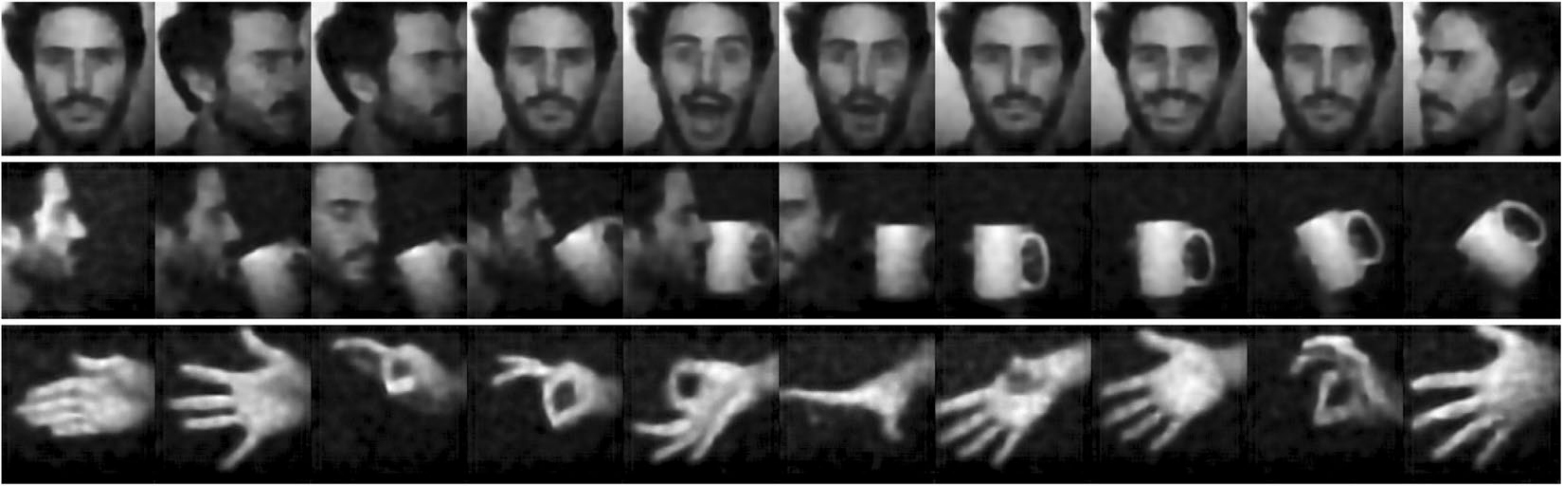
A selection of single-pixel camera image frames. These images (pixel size 128 × 128) are sampled using the deep learned binary sampling basis (*M* = 666, 4% of number of pixels) and reconstructed using the deep learning reconstruction algorithm.

A sample of different images reconstructed using the deep learning algorithm are shown in Fig. 6. It is important to emphasise that the image classes in the training data did not include faces, hands or household objects such as mugs. Subsequent investigations could offer better performance when the training data is task-specific.

## Discussion

Here we propose and test a novel technique for optimising low dimension feature identification with binary weights and reconstruction using a deep convolutional auto-encoder network. The method deals simultaneously with feature identification and reconstruction. It incorporates a new regularization approach that encourages binary weights, with the goal being to use the proven performance of continuous deep neural networks to learn bases in order to represent images efficiently. The alternative approach of compressive sensing is based on the assumption that a small collection of non-adaptive linear measurements of a compressible signal or image contain enough information for reconstruction and processing. At high levels of compression this may not be the case. We use deep learning to solve the inverse problem and reconstruct the image but also to learn an optimised measurement basis appropriate to the scene. We can think of the optimised sampling basis used in this approach as having learned the features common in the training library, without being defined explicitly by the user. In addition the compressive sensing solution is iterative and computationally costly whereas the deep learning solution is fast and deterministic.

We implemented the learned binary basis for sampling on a single-pixel camera, and in conjunction with the DCAN reconstruction pipeline. We found that the use of deep learning improves the image reconstruction quality of single-pixel cameras employing compressive sensing for moderate resolutions between 32 × 32 pixels and 128 × 128 pixels, and importantly this is achieved at video frame rates in excess of 30 Hz. Our objective was to find a balance between performance and rate of reconstruction to achieve high resolution in real-time. The intention of this work was a proof-of-principle demonstration for deep learning applied to image reconstruction from a single-pixel camera. Increasing depth by adding more layers can improve accuracy but adds to the reconstruction time. Future work will look at ways of resolving this issue, such as mimicking deep layers with shallow layers, to improve both reconstruction performance and rate. Further improvements are expected through extending training to larger datasets and faster GPU units, and in context-specific bases trained on specific image collections. This work represents a significant step towards real-time applications of computational imagers, and opens up possibilities for task-specific adaptation, with importance for applications such as gas sensing, 3D imaging and metrology.

## Methods

### Hadamard basis and the fast Hadamard transform

**Figure 7:**
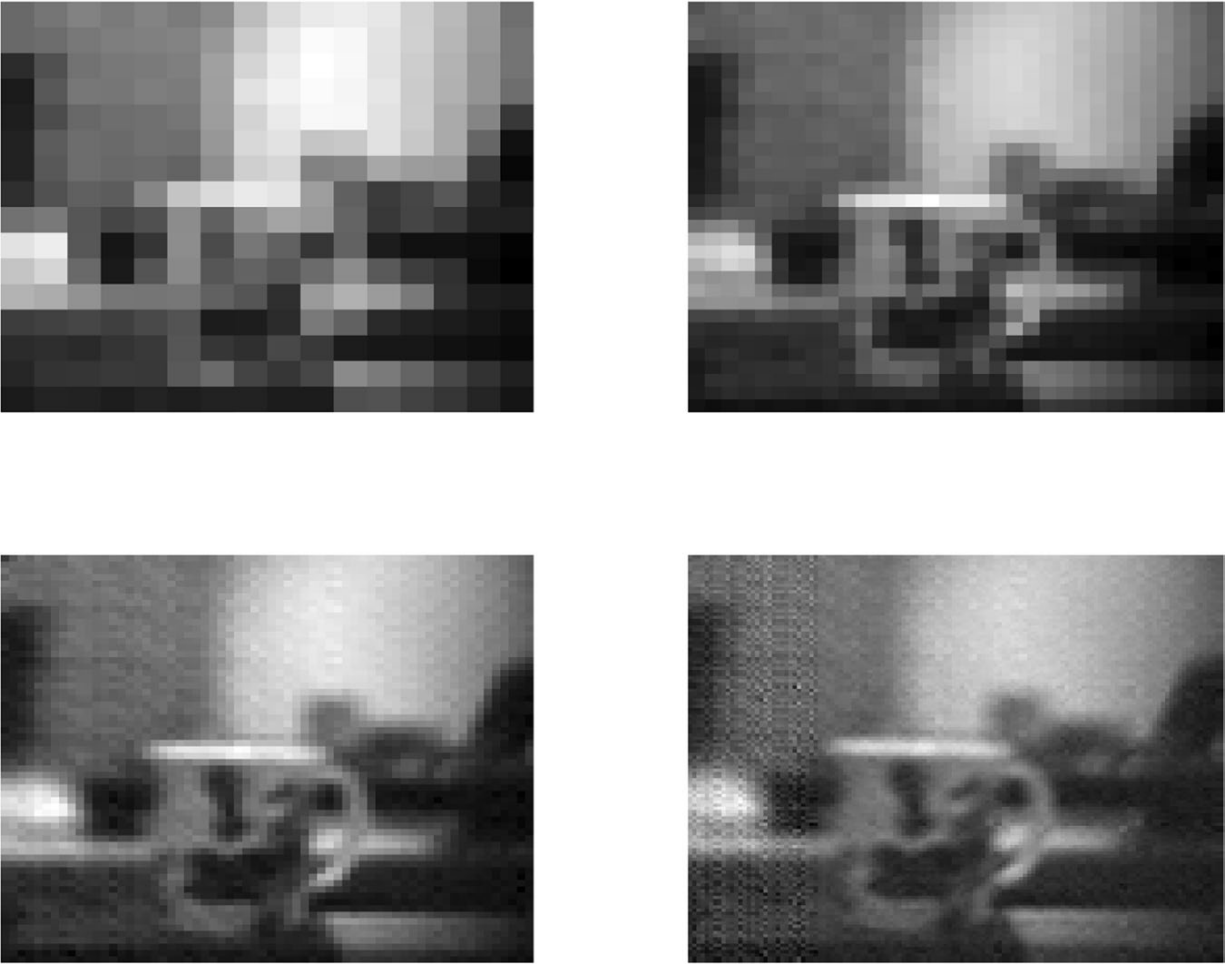
Signal-to-noise ratios at different resolutions. At 16 × 16 (SNR = 27.5) *top left*, at 32 × 32 (SNR = 14.2) *top right*, at 64 × 64 (SNR = 6.7) *bottom left* and at 128 × 128 (SNR = 3.4) *bottom right*.

The Hadamard basis is a set of *n* × *n* orthonormal binary matrices with *n* rows and *n* columns. The inverse of each element, *k*, of the Hadamard basis, **H**_*k*_, is its transpose, ${{\bf{H}}}_{k}^{{\rm T}}$, so images can be reconstructed perfectly from a clean signal. For an *n* × *n* image, **x**, the signal element, obtained from applying one element or pattern from the Hadamard basis, is *y*_*k*_ = H_*k*_**x**, and *x*_*k*_ can be recovered by applying the inverse Hadamard, ${x}_{k}={{\bf{H}}}_{{\rm{k}}}^{{\rm{{\rm T}}}}{\bf{y}}$ to the full signal **y**. However, when the signal is corrupted with detector noise, *ε*, the reconstruction, ${{\bf{H}}}_{{\rm{k}}}^{{\rm{T}}}({\bf{y}}+\varepsilon )$, is confounded by noise levels which can dominate signal elements with small absolute values. For a noisy signal, the inverse Hadamard transform disperses the noise over the reconstructed image, improving the signal-to-noise-ratio (SNR). Even so, the SNR approximately halves with each four-fold increase in resolution, see Fig. 7, causing issues for high resolution images.

The structure of the Hadamard basis and the fact that the Hadamard has binary values of ±1 means that each pixel of the image can be reconstructed using only addition or subtraction of the measured signals, see^12^ for further explanation. Hence, the Hadamard transform for an *N* = *n* × *n* image can be computed in *N*log_2_*N* addition/subtraction operations instead of order *N*^2^ floating point operations required for a general matrix, using the fast Hadamard transform algorithm decribed in^12^, as well as avoiding storage of the Hadamard basis elements (a substantial memory requirement for images 128 × 128 and above).

### Optimising the reduced Hadamard basis

Having established in the previous section that reconstruction is confounded by noisy signals with a low absolute value, we propose to exploit this observation to reduce the number of signals and to improve SNR by identifying and removing the basis elements most likely to have low absolute values. First we apply the Hadamard basis to a large number of 20,000 images^16^ to obtain simulated signals for each image, **y**^*i*^. We then order the basis elements in descending absolute value of their coefficients in the sum of signals ${\sum }_{i\mathrm{=1}}^{i\mathrm{=20,000}}{{\bf{y}}}^{i}$. In this way we identify the signal elements or pattern number that contribute, on average, the most to the reconstructed images. We denote this order as the optimised Hadamard ordering (*OH*_1…*N*_). To reconstruct an image ${x}_{k}^{r}={{\bf{H}}}_{k}^{{\rm{{\rm T}}}}{{\bf{y}}}^{r}$ from *M* signals, we take the first *M* signal elements from the optimised Hadamard ordering and set all other signal elements to zero

1 $${y}_{k}^{r}=(\begin{array}{cc}{y}_{k}, & {\rm{if}}\,k\in O{H}_{1\ldots M}\\ \mathrm{0,} & {\rm{otherwise}}.\end{array}$$

### Deep Learning for Single-Pixel Cameras

Deep learning is a set of tools that have become popular in a wide range of applications. There are many sophisticated software packages in the public domain. We have used MATCONVNET running on a Tesla C2075 GPU unit to create a deep convolutional auto-encoder as justified in^21^. The objective of our network is to replicate an input scene as an output image with resolution size i × *n*. The network is predefined in terms of number of layers, type of layers, the size and number of associated filters. The resolution of the output image, in terms of size, is dependent on the dimensions of the encoding filters and first decoding filters. In terms of quality, there are several factors: the number of encoding filters but also the structure of the DCAN. The layers, described below, are designed with this objective in mind. Code for the DCAN is provided in the supplementary file. The parameters of the network are optimised by training with a large dataset and minimising a Euclidean loss function using stochastic gradient descent^22^. We base our DCAN super resolution decoding architecture on^11^ but the encoding architecture is adapted to mimic the measurement of light intensity by the DMD. As illustrated and described in Fig. 2, the DCAN can be visualised as a series of filters comprising encoding and decoding layers, which map input features to output features. The encoding layers collectively encode the image and the decoding layers collectively decode the signal. The resolution of the output image, in terms of size, is dependent on the dimensions of the encoding filters (DMD patterns) and its corresponding decoding filter. In terms of quality, there are several factors: the number of encoding filters (DMD patterns) and also the structure of the DCAN.

#### Encoding Layer

The first layer comprises *M* filters (patterns) with height and width dimensions *n* × *n* that sequentially sample the scene and map to a feature vector of length M. In order for the learnt weights of this dense mapping to be suitable for the DMD, these weights are gradually regularised to take the binary values −1, 1. In this work, we propose *M* filters (patterns) of 333 and 666 which, in terms of signal acquisition, would allow video rate reconstruction at 30 Hz and 15 Hz respectively. For the filter resolutions we consider 32 × 32, 64 × 64 and 128 × 128.

#### Decoding Layers

The first decoding layer is a fully connected layer that takes the M dimension feature vector as its input and outputs a feature vector with *n* × *n* resolution. For training the DCAN using the image library, we added Gaussian noise which simulates the level of experimental noise and is also found to help regularise the solution. Inserted after the encoding and first decoding layer is a batch normalising layer^23^ which we find speeds up training. For the subsequent decoding layers, we add the following, with the aim of improving the resolution inspired by^11^: a convolutional layer with weight dimensions [9,9,1,64]; a rectified linear unit layer (RELU); a convolutional layer with weight dimensions [1,1,64,32]; RELU; a convolutional layer with weight dimensions [5,5,32.1] and a final RELU. The RELU layers add a piecewise linear non-linearity which avoids the ‘disappearing gradient’ problem when differentiated^24^. The cost function is least squares Euclidean distance. In the examples shown in this paper, we are working with image output resolutions of 32 × 32, 64 × 64 and 128 × 128.

### Data and training

The STL-10 dataset^16^ is an image recognition dataset (100,000 images comprising 10 classes: airplane, bird, car, cat, deer, dog, horse, monkey, ship, truck) for developing unsupervised feature learning, deep learning, self-taught learning algorithms. We use the same dataset for all our deep learning at different resolution experiments but resize the images from 96 × 96 to 32 × 32, 64 × 64 and 128 × 128. In our tests, images are further converted to gray-scale to match the measurement basis of the single-pixel camera. We set aside 10,000 images for validation and testing and train on the remaining 90,000 images.

Creating the DCAN and training was carried out using MATCONVNET^25^ running on a Tesla C2075 GPU unit.

### Using deep learning to derive optimal binary basis

To drive the weights towards binary values, we propose and test the following scheme, denoted ${{\rm{\Omega }}}_{\{-\mathrm{1,1}\}}$. The idea is to encourage the weights of the *bottleneck layer* towards {−1, 1}, where −1 represents ‘off’ and 1 represents ‘on’ on the DMD. The negative values are obtained post sensing by subtracting the measurement from the inverse mask. The proposed expression for this binary weight regularization scheme is

2 $${{\rm{\Omega }}}_{\{-\mathrm{1,1}\}}=\sum _{l}^{L}\sum _{j}^{n}\sum _{i}^{k}{(1+{w}_{ij}^{l})}^{2}{(1-{w}_{ij}^{l})}^{2},$$

where *w*_*ij*_, *l*, *j* and *i* denote the weight, the bottleneck layer and the weights’ width and height positions respectively.

We found that regularising the weights of the bottleneck layer to take binary values and control a noisy signal too quickly locked the potential of the decoding layers. We therefore propose training in two stages. First, the DCAN is trained without noise and binary regularisation to obtain an ideal decoder. Second, the parameters associated with the decoder layers are fixed and the encoding layers are retrained with noise and a low binary regularization (10:90 weighting with the cost function). Another advantage of this approach is that we obtain an ideal decoder that could be reused with an application-specific encoding layer.

### Single-pixel camera

The single-pixel camera is an optical computer that sequentially measures the inner products between an *N* × *N* pixel sampled version *x* of the incident light-field from the scene under view and a set of *M* ≤ *N*^2^ two-dimensional *N* × *N* binary masks or patterns, collectively called the measurement basis.

An image of the scene is formed on to a DMD consisting of an array of 1024 × 768 mirrors^2^. Each mirror or subset of mirrors corresponds to a particular pixel in *x* and the mask, and can be independently orientated either towards another biconvex lens (corresponding to a one at that pixel in the basis) or away from that lens (corresponding to a zero at that pixel in the basis). The reflected light is collected by the second lens and focused onto a single photon detector (the single pixel) that integrates the product between *x* and each basis to compute the measurement *y* as its output voltage. This voltage is then digitized by an A/D converter, see Fig. 1. A measurement basis that uses ±1 can be employed by taking a differential measurement between each mask and its inverse. This approach is advantageous to signal-to-noise ratio^5^. Measurement bases such as Walsh-Hadamard and Fourier enable efficient reconstruction through fast algorithmic transform implementations but used in full require *M* = *N*_2_ masks or patterns. In order to obtain high resolution images at video rate we require *M* ≪ *N*^2^.

### Compressive sensing

Recovering an image, *x*, from a set of under-sampled measurements, *y*, is formulated as an inverse problem y = Φ*x* where Φ is a linear measurement basis. Compressive sensing seeks to solve the linear inverse problem in the case where *x* has a sparse representation, and is based on the assumption that a small collection of non-adaptive linear measurements of a compressible signal or image contain enough information for reconstruction and processing^17–19^.

## Electronic supplementary material

Supplementary Materials
