# Supplementary material for: Deep learning for real-time single-pixel video
Source: Sci Rep. 2018 Feb 5;8:2369. doi: 10.1038/s41598-018-20521-y (PMC5799195; doi:10.1038/s41598-018-20521-y)
Supplement: Supplementary file 1 — Supplementary Materials [file 41598_2018_20521_MOESM1_ESM.pdf]

SUPPLEMENTARY MATERIALS

Deep learning for real-time single-pixel video.

Catherine F. Higham[1], Roderick Murray-Smith[1], Miles J. Padgett[2] and Matthew P. Edgar[2*]

[1]*School of Computing Science, University of Glasgow, Glasgow, Glasgow, G12 8QQ, UK and*

[2]*School of Physics and Astronomy, University of Glasgow, Glasgow, G12 8QQ, UK*

* catherine.higham@glasgow.ac.uk

I. EXAMPLE: MATLAB SCRIPT.

```
% ------------------------------------------------------------------------
function net = getSTL10AutoencoderNet
% ------------------------------------------------------------------------
lr = [0.001 0.001] ;
net.layers = {} ;

Lb=333;%set desired number of patterns
CL=32^2;%set desired resolution for the DMD

%Encoding Fully Connected Block
%Layer 1
net.layers{end+1} = struct('biases'          , zeros(1, Lb, 'single')           , ...
                     'biasesLearningRate' , 0                                , ...
                     'biasesWeightDecay'  , 0                                , ...
                     'filters'            , sparse_initialization([1 1 CL Lb])   , ...
                     'filtersLearningRate', 1                                , ...
                     'filtersWeightDecay' , 1                                , ...
                     'name'               , 'binary_1'                       , ...
                     'pad'                , [0 0 0 0]                        , ...
                     'stride'             , [1 1]                            , ...
                     'type'               , 'conv'                           );
%Layer 2
net.layers{end+1} = struct('type', 'addnoise',   'sigma', 0) ;
%Layer 3
net.layers{end+1} = struct('type', 'bnorm', ...
  'weights', {{ones(Lb, 1, 'single'), zeros(Lb, 1, 'single'), zeros(Lb, 2, 'single')}}, ...
  'learningRate', [2 1 0.05], ...
  'weightDecay', [0 0]) ;

%Decoding Fully Connected Block
%Layer 4
net.layers{end+1} = struct('biases'          , zeros(1, CL, 'single')        , ...
                     'biasesLearningRate' , 0                                , ...
                     'biasesWeightDecay'  , 0                                , ...
                     'filters'            , sparse_initialization([1 1 Lb CL])   , ...
                     'filtersLearningRate', 1                                , ...
                     'filtersWeightDecay' , 1                                , ...
                     'name'               , 'encoder_1'                      , ...
                     'pad'                , [0 0 0 0]                        , ...
                     'stride'             , [1 1]                            , ...
                     'type'               , 'conv'                           );

 %Layer 5
 net.layers{end+1} = struct('type', 'bnorm', ...
   'weights', {{ones(CL, 1, 'single'), zeros(CL, 1, 'single'), zeros(CL, 2, 'single')}}, ...
   'learningRate', [2 1 0.05], ...
   'weightDecay', [0 0]) ;

%Layer 6
 net.layers{end+1} = struct('type', 'reshape', 'NP', 32);%reshape N pixels N^0.5 x N^0.5 pixels

% Convolutional Block 1
%Layer 7
net.layers{end+1} = struct('type', 'conv', ...
                           'weights', {{0.01*randn(5,5,1,64, 'single'), zeros(1, 64, 'single')}}, ...
                           'learningRate', lr, ...
                           'stride', 1, ...
                           'pad', 2) ;
%Layer 8
net.layers{end+1} = struct('type', 'relu');
```

```
% Convolutional Block 2
%Layer 9
net.layers{end+1} = struct('type', 'conv', ...
                           'weights', {{0.01*randn(1,1,64,32, 'single'), zeros(1, 32, 'single')}}, ...
                           'learningRate', lr, ...
                           'stride', 1, ...
                           'pad', 0) ;
%Layer 10
net.layers{end+1} = struct('type', 'relu');

% Convolutional Block 3
%Layer 11
net.layers{end+1} = struct('type', 'conv', ...
                           'weights', {{0.01*randn(5,5,32,1, 'single'), zeros(1, 1, 'single')}}, ...
                           'learningRate', lr, ...
                           'stride', 1, ...
                           'pad', 2) ;
%Layer 12
net.layers{end+1} = struct('type', 'relu');

%Euclidean Loss
%Layer 13
net.layers{end+1} = struct('type', 'euclideanloss');



end
```
